# Transdermal Delivery of Scopolamine by Natural Submicron Injectors: *In-Vivo* Study in Pig

**DOI:** 10.1371/journal.pone.0031922

**Published:** 2012-02-21

**Authors:** Esther Shaoul, Ari Ayalon, Yossi Tal, Tamar Lotan

**Affiliations:** 1 NanoCyte (Israel) Ltd, Caesarea, Israel; 2 Marine Biology Department, The Leon H. Charney School of Marine Sciences, University of Haifa, Haifa, Israel; University of California, Merced, United States of America

## Abstract

Transdermal drug delivery has made a notable contribution to medical practice, but has yet to fully achieve its potential as an alternative to oral delivery and hypodermic injections. While transdermal delivery systems would appear to provide an attractive solution for local and systemic drug delivery, only a limited number of drugs can be delivered through the outer layer of the skin. The most difficult to deliver in this way are hydrophilic drugs. The aquatic phylum Cnidaria, which includes sea anemones, corals, jellyfish and hydra, is one of the most ancient multicellular phyla that possess **s**tinging cells containing organelles (cnidocysts), comprising a sophisticated injection system. The apparatus is folded within collagenous microcapsules and upon activation injects a thin tubule that immediately penetrates the prey and delivers its contents. Here we show that this natural microscopic injection system can be adapted for systemic transdermal drug delivery once it is isolated from the cells and uploaded with the drug. Using a topically applied gel containing isolated natural sea anemone injectors and the muscarinic receptor antagonist scopolamine, we found that the formulated injectors could penetrate porcine skin and immediately deliver this hydrophilic drug. An *in-vivo* study in pigs demonstrated, for the first time, rapid systemic delivery of scopolamine, with T_max_ of 30 minutes and C_max_ 5 times higher than in controls treated topically with a scopolamine-containing gel without cnidocysts. The ability of the formulated natural injection system to penetrate a barrier as thick as the skin and systemically deliver an exogenous compound presents an intriguing and attractive alternative for hydrophilic transdermal drug delivery.

## Introduction

Transdermal delivery has advantages over conventional drug delivery systems in that it reduces first-pass metabolism, eliminates drastic drug fluctuations, prevents adverse side effects, and increases patient compliance. A variety of vehicles and technologies have been developed to penetrate the skin barrier [1], allowing transcutaneous drug delivery [2], [3]. The two main approaches employed to augment skin permeability and penetration are based on utilization of passive chemical enhancers or advanced active physical energetic devices. The passive approach includes the use of different types of topical formulations, including chemical enhancers, liposomes, ethosomes, or large reservoir-type patches [4], [5]. However, while these technologies have the advantage of high patient compliance, they usually require a prolonged application time and are limited to molecules that are both lipophilic and small. The physical approach primarily employs high-energy devices such as needle-free injectors, low-frequency ultrasound, iontophoresis, electroporation, microneedles, or thermal procedures [6], [7]. These advanced methods have the advantage of rapidly permeating the skin barrier, but they generally entail sophisticated devices, professional administration, higher costs, and lower patient compliance. Owing to these limitations relatively few drugs can currently be introduced transdermally, with particular difficulty experienced in the delivery of hydrophilic drugs through this route [8].

**Figure 1:**
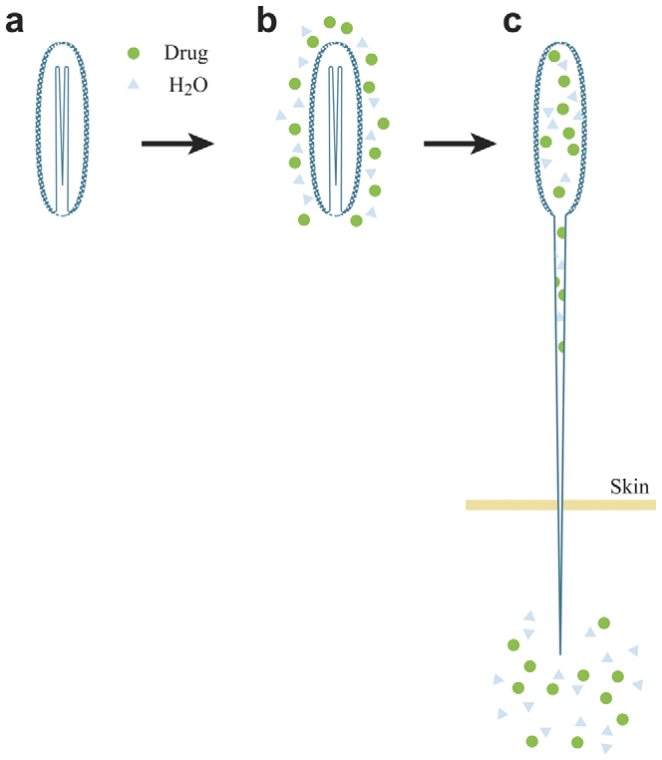
Mode of action of the cnidocyst system. The intact dry cnidocyst (a) is activated by the hydrophilic drug solution (b) water molecules (blue triangle) and the soluble drug (green circles) penetrate the porous wal**l** of the cnidocyst, resulting in discharge of the tubule and injection of its microcapsule content through the tubule to the skin (c).

The phylum Cnidaria, dated to around 700 million years ago, typically features stinging cells containing cnidocysts, microcapsules equipped with a submicron injection system [9], [10]. Activation of the microcapsule engenders a high internal pressure of 150 bars, resulting in the discharge of a long folded tubule at an ultrafast acceleration of 5×10^6^ g [11], [12]. About 30 subtypes of cnidocysts are known. They differ in size and shape, but all function according to the same basic principle (Fig. 1). Discharge of the system is controlled by osmotic balance. Once the system is activated, water flows through the porous cnidocyst wall causing dissociation of a large aggregated matrix of poly-γ-glutamate trapped by cationic metal. This in turn increases the osmotic pressure, resulting in ejection of the tubule [12], [13], [14]. The poly-γ-glutamate matrix serves as the internal “battery” of the system and this, together with the high tensile strength of the cnidocyst wall [15], can build up the pressure required to propel the tubule out at an acceleration high enough to puncture the skin of the targeted prey and immediately deliver the cnidocyst content [16]. We have previously shown that the sea anemone micro-injection system can be applied for topical delivery, is compatible with hydrophilic compounds, and is clinically safe [16], [17], [18].

The aim of the present study was to evaluate the potential use of the cnidocyst system as an active vehicle for systemic drug delivery. The drug of choice was scopolamine, as its delivery profile in topical applications is well documented. The animal model we chose was the pig, as it is similar to humans in its anatomy, genetics, physiology and biochemistry, and is well recognized as an experimental animal in biomedical research [19], [20]. It is a particularly reliable model for transdermal research *in vivo* because its skin is highly similar to human skin in having low hairiness, a thick strateum corneum, and similar dermis composition, as comprehensively reviewed by Simon and Maibach [21]. Using a porcine model, we compared the pharmacokinetic profile of scopolamine following a single topical application of the cnidocyst system to that of a control topical application without cnidocysts.

## Materials and Methods

### Ethics Statement

All animals in the study were handled according to the guidelines for efficacy testing of veterinary medical products (Rules Governing Medicinal Products in the European Union, Volume 7). The experiment protocol was approved by the Animal Care Committee of the Experimental Institute of the Veterinary Faculty of the Szent István University, Hungary. The ID number was PCG-206.

### Chemicals and Formulations

Scopolamine hydrobromide solution (scopolamine HBr) and all reagents were purchased from Sigma-Aldrich (Israel) unless stated otherwise, and were of at least analytical grade. Acetonitrile (HPLC grade) and ethanol were purchased from Riedel de Haen (Seelze, Germany). Klucel® HF Pharm hydroxypropylcellulose (HPC) was received from Hercules Aqualon (Wilmington, DE, USA). The control gel formulation contained 2% HPC in absolute ethanol. The cnidocyst gel contained 0.075×10^6^ microcapsules/mg of the above control gel, and was applied to the skin in a formulation of 1×10^6^ microcapsules/cm^2^.

### Preparation of microcapsules

**Figure 2:**
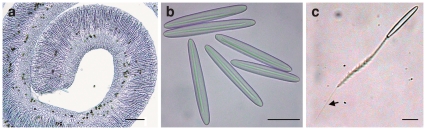
Preparation of cnidocysts. (a) A filament of *Aiptasia diaphana* containing packed cnidocysts. Bar, 100 µm. (b) Isolated cnidocysts. Bar, 25 µm. (c), A discharging cnidocyst. Following release of the folded 150-µm injector, only its smooth tip (50 µm long, submicron diameter) can penetrate the skin (see arrow). Bar, 25 µm.

The sea anemone *Aiptasia diaphana*, when triggered mechanically, secretes thin filaments (acontia) enriched with microcapsules (Fig. 2). We exploited this natural behavior to obtain filaments without harming the anemone. Microcapsules were isolated from the filamentous tissue as described previously [16], [22], [23]. Briefly, filaments were incubated in 1 M sodium citrate, followed by two centrifugations in 70% Percoll gradients. The isolated microcapsules were washed with decreasing concentrations of NaCl (1 M to 0.2 M) and freeze-dried. The purified microcapsules were kept as a lyophilized powder until use.

### *In-vitro* Permeability Tests

#### Tape-stripping assay

Isolated porcine ear skins were covered with cnidocyst gel, and 0.05% toluidine blue dye solution was then added. After 5 minutes the treated sites were rinsed with distilled water, gently wiped to remove the applied materials, and photographed. To examine dye permeability the skin was tape-stripped 30 times to remove the stratum corneum and the residual skin was photographed under a Zeiss Axioskop 40 microscope.

#### Franz diffusion cell and HPLC analysis

Permeability of scopolamine HBr 5% solution through the full skin of 8-week-old female nude mice (CD1 strain) was measured *in vitro* in a Franz diffusion cell system as previously described [16]. In brief, cnidocyst gel (8 mg) was introduced into the donor chambers on the skin and overlaid with excess of scopolamine HBr 5% solution. As a control, skin pieces were covered with 8 mg of gel without cnidocysts, and overlaid with the same scopolamine HBr solution. After 5 minutes the scopolamine HBr solution was removed and the embedded skins in the Franz cell apparatus were thoroughly washed 5 times with Millipore DDW. The skins were then left in the diffusion cell for up to 24 h to allow the delivered drug to diffuse under the skin. During that period, samples (2 ml) were taken from the receiver compartment after 0.5, 1, 1.5, 3, 5, and 23–24 h, and after each withdrawal the receiver was filled with fresh saline solution. The samples were analyzed by HPLC (Hewlett Packard LC1090 Liquid Chromatograph with a diode-array detector). The analysis was performed at room temperature using a 5-mm Kromasil Cyano column (KR60-5CN; 250×4.6 mm). The mobile phase consisted of 68% (A) 20% acetonitrile in 20 mM, pH 6 phosphate buffer, and, 32% (B) Acetonitrile. The flow rate was 1.2 ml/min and the UV detector was set at a wavelength of 210 nm. The injection volume was 40 µl and scopolamine retention time was 7.5 minutes [24], [25]. Data were expressed as the cumulative scopolamine permeation per square centimetre of skin surface.

### *In-Vivo* Study in Pigs

Seven white Landrance pigs, each weighing approximately 20 kg, were sedated with ketamine (100 mg/ml) and medetomidine (Domitor®, Pfizer; 1 mg/ml). The animals were injected intramuscularly with a combination of these two drugs (0.6–0.7 ml/kg body weight), giving approximately 30 minutes of sedation. Four pigs (labelled 4–7A) were topically treated with the cnidocyst gel containing 1×10^6^ cnidocysts/cm^2^ and 5% scopolamine HBr, and three with the control gel (without cnidocysts) (labelled 1–3A) and 5% scopolamine HBr. The back of each animal was gently shaved 24 h before the start of the experiment. A 15-cm^2^ application area on the shaved back was then rinsed with 70% ethanol, and a foam ring was applied to limit the application site. Test or control gel preparations (190 mg) were applied, followed by immediate application of excess of scopolamine HBr. After 5 minutes the treated sites were washed with distilled water and gently wiped to remove the applied product.

From each pig, 12 blood samples were obtained via the antebrachial cephalic sinus. The first sample was obtained immediately before the topical dose application and the rest were collected 0.5, 1, 1.5, 2, 3, 4, 6, 8, 10, 12 and 24 h afterwards. Scopolamine concentrations were assayed using a validated liquid chromatography–mass spectrometry (LC–MS/MS(+)) method with a lower limit of quantification of 10.4 ng/l [26]_ENREF_15_ENREF_15._ENREF_15_ENREF_15 The HPLC system was equipped with a Merck LiChrospher 100 RP select B, 5 µm (4×75 mm) column and an MS/MS Sciex API 4000 detector. Pharmacokinetic evaluations of C_max_, T_max_, half-life elimination time (t_1/2_) and area under the concentration curve (AUC) were calculated using WinNonlin (Pharsight) pharmacokinetic software. No abnormal or adverse physiological effects were observed in any of the animals.

## Results

### Preparation of Cnidocysts for Drug Delivery

We used the naturally secreted acontial filaments of the sea anemone *Aiptasia diaphana*, which are highly enriched with cnidocysts (Fig. 2a). Cnidocysts were isolated from the filaments by exploiting their high density and high stability in salts, and were then freeze-dried. An isolated microcapsule preparation contained one type of intact cylindrical microcapsule, 60 µm in length and 8 µm in diameter, with a 150-µm-long injector folded within (Fig. 2b). The injector is composed of two main parts: a 100-µm-long shaft containing barbs and a 50-µm-long smooth tubule with a diameter smaller than 1 µm (Fig. 2c). The isolated preparation was kept dry and did not lose its potential discharge characteristics [16]. Prior to its application over the skin the preparation was formulated into an anhydrous gel consisting of 2% hydroxyl propyl cellulose in absolute ethanol. This gel formulation kept the cnidocysts in close contact with the skin, but did not trigger their discharge. Activation of the microcapsules occurred only when they were exposed to the hydrophilic solution.

### Immediate Delivery to Viable Epidermis

**Figure 3:**
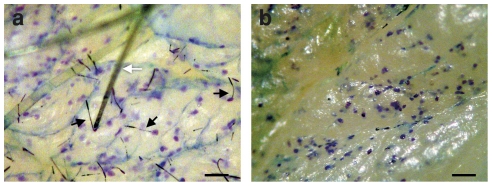
*In-vitro* delivery of hydrophilic compounds by cnidocysts in a gel formulation. (a) Activation of cnidocyst gel over isolated porcine ear skin with 0.05% toluidine blue dye solution for 5 min. Following application the skin was thoroughly washed and then photographed. Black arrows, penetrating cnidocyst. White arrow, skin hair bundle. Scale bar, 100 µm. (b) The skin was tape-stripped 30 times to remove the stratum corneum and then photographed. Scale bar, 100 µm.

To determine whether the isolated cnidocysts could penetrate porcine skin and deliver an exogenous hydrophilic compound to the viable epidermis, the cnidocyst gel was applied *in vitro* over an isolated piece of porcine skin and the preparation was activated with a hydrophilic solution containing 0.05% toluidine blue dye solution. Five minutes after activation the skin was thoroughly washed and the stratum corneum was removed (Fig. 3a and b). The results showed that the isolated cnidocysts had penetrated the stratum corneum and delivered the dye to the viable epidermis within 5 minutes of activation. Thus, the isolated system had retained its natural properties and the rehydration had been sufficient to trigger its activation.

### *In-Vitro* Delivery of Scopolamine

**Figure 4:**
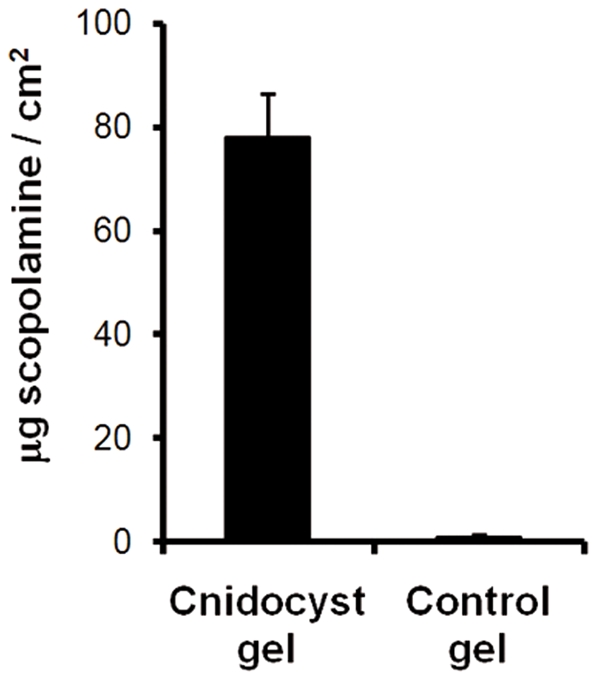
Scopolamine 5-minute *in-vitro* study. Cumulative permeation of scopolamine delivered by cnidocyst gel (*n* = 11) compared to control gel without microcapsules (*n* = 5) after 24 hours. Error bars represent s.d.

To obtain quantitative results of a short application of the cnidocyst gel, we used the Franz diffusion cell system to measure the amount of the potent muscarinic receptor antagonist scopolamine delivered *in vitro* through the full-thickness skin of a nude mouse. We chose this preparation as it is a convenient and readily available model for percutaneous penetration [27], and has been found to serve as a suitable skin model for testing cnidocyst system quantitative delivery [16]. The cnidocyst gel was applied for 5 minutes on the skin with 5% scopolamine as in the dye experiment. HPLC analysis revealed rapid and active drug delivery by the cnidocyst gel (Fig. 4). The amount of scopolamine delivered to the skin was 78.2±27 µg/cm^2^, whereas in control skin samples that received gel without cnidocysts the amount of scopolamine delivered was negligible (0.99±1.1 µg/cm^2^).

### Pharmacokinetics of Scopolamine

**Figure 5:**
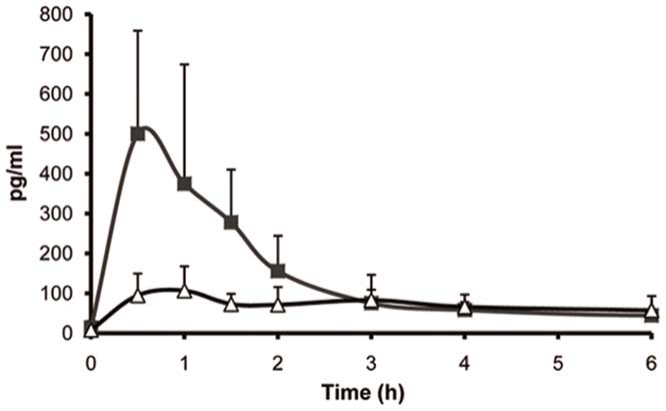
Plasma levels of scopolamine in pigs after short topical exposure. Test and control groups were exposed for 5 minutes to the same solution of 5% scopolamine HBr. Averaged values (means ± s.d) of the test group (▪) treated with gel formulation containing isolated cnidocysts and of the control group (Δ) treated with gel formulation only.

The ability of the natural injectors to deliver scopolamine *in vivo* was tested in a porcine model. The experiment was performed on seven pigs, four of which (experimental group) were treated with the topically applied gel containing cnidocysts, while the other three (control group) received the gel without cnidocysts. Both groups were exposed to 5% scopolamine solution for only 5 minutes and 12 blood samples were collected up to 24 hours. Table 1 presents the scopolamine pharmacokinetic parameters calculated from the blood samples collected from each pig, reflecting the ability of the natural injectors to deliver scopolamine systemically. Peak plasma concentrations (C_max_) were up to five times higher, on average, in the experimental group than in the control group (500±258 pg/ml compared to 133±31 pg/ml; Fig. 5). In addition, the time to maximal concentration (T_max_) was significantly shorter in the experimental group than in the control (0.5±0 h compared to 2.7±1.5 h), and the average area under the concentration curve (AUC_0–24_), representing the mean drug concentration in the body, was nearly two fold higher for the experimental group than for the control. No adverse or abnormal effects were observed in the pigs during the experimental procedure. Thus, although the drug delivery route was topical, the injection system tested in this study had a relatively short onset time and enabled the drug to accumulate rapidly in the plasma.

**Table 1:** Calculated pharmacokinetic parameters of scopolamine in pig plasma after a 5-minute topical application of the drug.

Parameter*	Units	Pig No.						
		Control group			Experimental group (Cnidocysts) Preparation			
		1A	2A	3A	4A	5A	6A	7A
Cmax	pg/ml	155	98.4	148.4	419.5	855.3	239.5	486.6
Tmax	hours	3.0	4.0	1.0	0.5	0.5	0.5	0.5
t_1/2_	hours	4.4	6.8	9.7	5.2	5.6	4.8	7.1
AUC_0–24_	hours(pg/ml)	1191.5	911.5	816.1	1973.0	2488.9	970.1	1155.3
AUC_INF_	hours(pg/ml)	1316.9	1028.7	946.5	2320.8	2628.7	1093.1	1289.9

* Cmax, peak plasma concentration; Tmax, time to maximal concentration; t_½_, half-life elimination time; AUC, Area under the plasma concentration time curve.

## Discussion

In this report we describe a naturally derived system, isolated from sea anemone, that operates as a submicron injection system and was used here as micro devices in topical application for delivery of the hydrophilic drug scopolamine. We showed that short (5 minutes) exposure of this system to hydrophilic compound is sufficient to facilitate delivery of the drug to viable pig epidermis. Successful systemic targeting of the topically applied scopolamine by this natural system *in vivo* was demonstrated here, for the first time, in a pharmacokinetic study that revealed a significantly shorter T_max_ (30 min) and a C_max_ up to 5 times higher than in the control.

Scopolamine, a naturally occurring alkaloid, is one of the most effective single agents used to prevent motion sickness [28] and is also commonly used for the prevention of postoperative nausea and vomiting [29]. Being a tertiary amine scopolamine hydrobromide diffuses passively through the blood-brain barrier; thus, its oral or parenteral administration results in enhanced severity of the drug's adverse effects, which may include visual disturbances, dizziness, agitation, dry mouth, hallucinations, amnesia and drowsiness [28]. Moreover, scopolamine intoxication is dose-dependent, and in addition to its short half-life in plasma, its oral or parenterally administration is more limited in clinical use [30]. Hence, the preferred mode of delivery is the transdermal route, which exhibits relatively low scopolamine toxicity manifested mainly with limited visual disturbances and dry mouth [29], [31]. Scopolamine was among the first drugs to be incorporated into a transdermal patch. When administered in that form, however, the drug does not exert its effect until 6 to 8 hours after patch application because of its characteristic slow, incremental transdermal diffusion [29], [32], [33]. Moreover, it was suggested that in order to achieve the desired concentration of scopolamine in the plasma, the patch system should be combined with oral administration [32], [34]. With the natural injection system described here, however, the drug was delivered immediately to the skin and the time taken to accumulation in the plasma was relatively short. Thus, despite its topical formulation, the pharmacokinetic characteristics of the drug were comparable to those reported for its subcutaneous injection [30]. Furthermore, topical application enjoys the additional advantages of ease of administration and hence better patient compliance. In this study we tested the effect of a single application of scopolamine using a selected microcapsule number and drug dose. We have previously shown, however, that the concentration of the delivered drug can be controlled by changing either the number of microcapsules or the drug concentration [16], [17]. Thus, when other drugs are used, these parameters need to be adjusted accordingly and additional doses added if necessary.

The natural injection system described here combines the benefits of chemical and physical transdermal technologies, both in the simplicity of its use and its rapid delivery mechanism. The technology that most closely resembles the cnidocyst delivery system is delivery by fabricated microneedles. Several sophisticated microneedle systems have been developed over the last decade [3], [7], [8], [35], [36], [37]. Most of them are drug-coated projections that pierce the skin to create pores, and in combination with a patch or other transdermal method, facilitate drug delivery. Others are active flow delivery devices composed of hollow needles. However, skin penetration by solid or hollow microneedles is critically dependent on the pressure applied to the microneedles array [3]. Thus for example, to achieve uniform pressure for insertion of the microneedle tip into the skin, application of the microneedle array requires that external energy be provided by an applicator device [35], [38], [39].

In contrast, the high energy needed to shoot a cnidocyst injector into the skin and pump the drug through the hollow tubule is a built-in feature of the isolated intact microcapsule. Therefore, no external energy is required and the microcapsules can be incorporated in a user-friendly gel that is easy to apply. Furthermore, safety trials conducted in more than 100 human volunteers demonstrated no irritation or allergenicity caused by the injection system [16], [18]. In addition, in a swine study, gentle wiping of the treatment site after application sufficed to remove the injectors from the skin, and in unwiped sites only traces of the injectors were found after 2 days [17]. The potential of the system lies also in its flexibility and modularity, since a wide range of different hydrophilic compounds can be attached to the basic gel containing the cnidocysts and used in different therapeutic contexts. Taken together, the above findings suggest that the natural injection technology offers an intriguing and attractive option for fast-acting hydrophilic transdermal drug delivery.
